# Nanosheet integration of induced tunnel field-effect transistor with lower cost and lower power

**DOI:** 10.1186/s11671-024-04036-2

**Published:** 2024-07-02

**Authors:** Jyi-Tsong Lin, Chia-Yo Kuo

**Affiliations:** Department of Electrical Engineering, National Sun Yat-Sen University, Kaohsiung, 80424 Taiwan, R.O.C.

**Keywords:** Nanosheet induced tunnel field-effect transistor (NS iTFET), Subthreshold swing (*SS*), Line tunneling, Band-to-band tunneling (BTBT), SiGe

## Abstract

Nanosheet transistors are poised to become the preferred choice for the next generation of smaller-sized devices in the future. To address the future demand for high-performance and low-power computing applications, this study proposes a nanosheet structure with a vertically stacked design, featuring a high *I*_ON_/*I*_OFF_ ratio. This Nanosheet design is combined with an induced tunnel field-effect transistor. By utilizing SiGe with a carrier mobility three times that of Si and employing a line tunneling mechanism, the research successfully achieves superior Band to Band characteristics, resulting in improved switching behavior and a lower Subthreshold Swing (*SS*). Comparative studies were conducted on three TFET types: Nanosheet PIN TFET, Nanosheet Schottky iTFET, and Fin iTFET. Results show that the Nanosheet PIN TFET has a higher *I*_ON_/*I*_OFF_ ratio but poorer SSavg values at 47.63 mV/dec compared to the others. However, with a SiGe Body thickness of 3 nm, both Nanosheet iTFET and Fin iTFET exhibit higher* I*_ON_/*I*_OFF_ ratios and superior SSavg values at 17.64 mV/dec. These findings suggest the potential of Nanosheet iTFET and Fin iTFET for low-power, lower thermal budgets, and fast-switching applications.

## Introduction

As Moore’s Law continues to evolve, the pursuit of faster switching speeds, lower power consumption, and smaller size variations has driven transistor technology through successive generations, transitioning from traditional planar transistor (Planar FET), nanowire, FinFET, to nanosheet. Among these advancements, nanosheets exhibit remarkable design flexibility, allowing the channel width to increase for enhanced current flow or decrease to limit power consumption. Stacked nanosheet transistors have been confirmed as the primary component structure for 3 nm technology nodes and smaller advanced technologies. Compared to FinFET, they demonstrate superior electrostatic characteristics and short channel control, making them the mainstream application for TSMC and Samsung in the 3 nm structure [1, 2].

However, Nanosheets also bring along significant challenges, such as the trade-offs between transistor switching speed, power consumption, process complexity, and cost. This trade-off is closely related to the channel width, commonly referred to as *W*_eff_*.* Larger widths imply the ability to drive more current, facilitating quicker transistor on–off transitions, but they also necessitate a more complex and expensive manufacturing process [3].

Despite Nanosheets becoming the mainstream application for TSMC and Samsung in the 3 nm architecture, MOSFETs still face challenges in overcoming the thermal limitation (thermal constraint) of 60 mV/decade *SS* at room temperature (300 K) and the difficulty in reducing the power supply voltage (V_D_) below 0.5 V [4]. As the Internet of Things (IoT) and artificial intelligence (AI) chip technologies rapidly advance, the increasing demand for higher voltages becomes an undeniable challenge for future device power consumption.

In response to this, researchers propose Tunnel Field-Effect Transistor (TFET) that leverage their Band-to-Band quantum tunneling mechanism to overcome carrier Boltzmann distribution. These devices, in comparison to Metal–Oxide–Semiconductor Field-Effect Transistors (MOSFETs), offer advantages, enabling high performance under extremely low operating bias conditions, achieving low power consumption and rapid switching effects [5, 6].

Normally, Tunnel Field-Effect Transistor (TFET) exhibits two tunneling current generation mechanisms [7]. The first is “point tunneling”, occurring at the source-channel interface, with its primary contribution confined to a small region. Due to the limited tunneling area, the Band-to-Band effect is restricted, resulting in a Subthreshold Swing (*SS*) that does not reach an ideal level. The second is “line tunneling”, located in the source region overlapping with the gate. As the region where Band to Band begins resembles a line, this component is referred to as “line tunneling”. Compared to point tunneling, line tunneling has a broader tunneling area, and the current is directly proportional to both the channel width (*W*) and channel length (*L*) of the device, effectively improving subthreshold swing (*SS*) [8–14]. In this work, we will discuss and compare the results obtained from these two tunneling mechanisms.

We have also made improvements in addressing the expensive manufacturing processes by adopting a SiGe (70% Si and 30% Ge) monolithic material for stacking. The source metal is configured as a Schottky contact, forming different Schottky barrier heights by utilizing metals with distinct work functions. This leads to the inversion of a thinner carrier inversion layer, replacing the need for doping and thermal annealing associated with traditional material stacking. Simultaneously, this approach expands the area on the source side, thereby increasing the linear tunneling area between the gate and source, further enhancing the device’s performance [5].

Our proposed iTFET utilizes Schottky contacts to achieve a total line-tunneling dominated TFET. In contrast to traditional TFETs that require doping to establish p-type and n-type regions for P–I–N or P–N–N structures, iTFETs use a single piece of N-substrate with uniform doping concentration. Due to the band bending, thermal activation creates an inversion layer, converting the Source region into P-type, thus forming an overall PN structure [15].

In Sect. 2, we will present the device design, manufacturing steps, and simulation methods. In Sect. 3 will discuss the circuit characteristics under various parameter variations and simulation results. Finally, Sect. 4 will summarize the conclusions of this study.

## Device design and simulation method

**Fig. 1:**
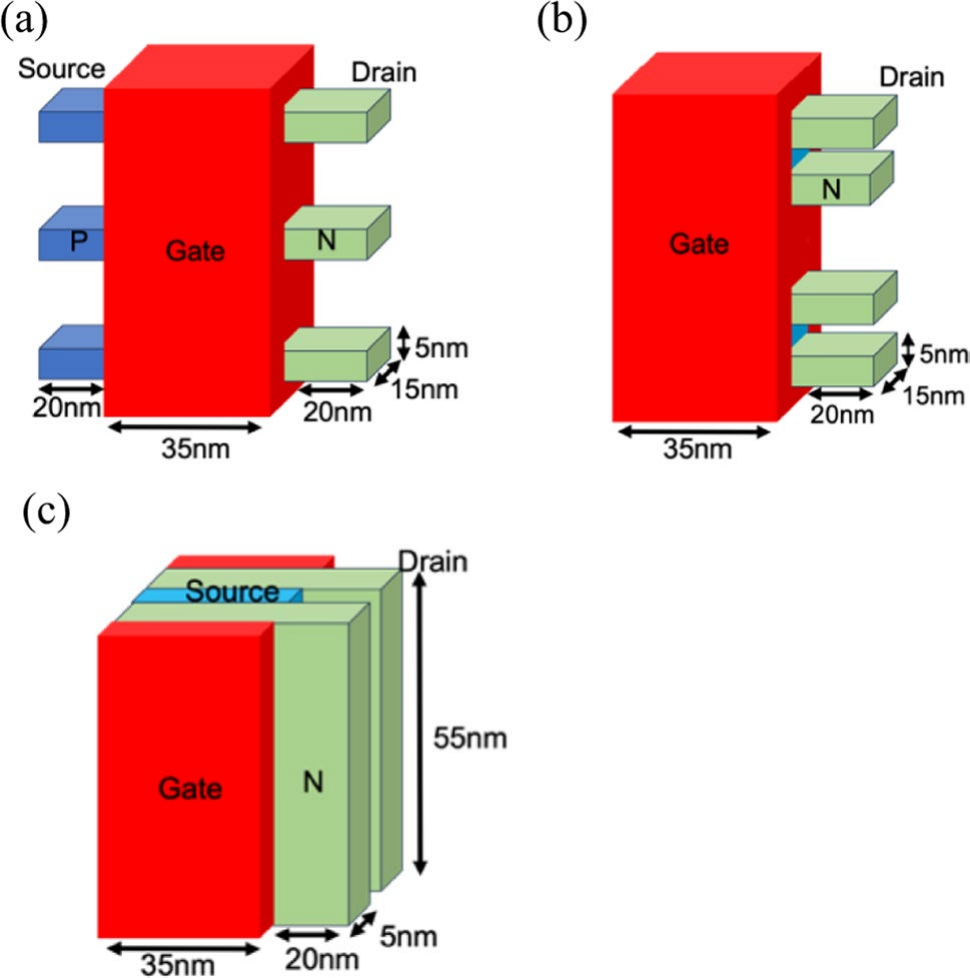
SiGe Body thickness is 5 nm of **a** Nanosheet PIN TFET. **b** Nanosheet iTFET. **c** Fin iTFET

**Fig. 2:**
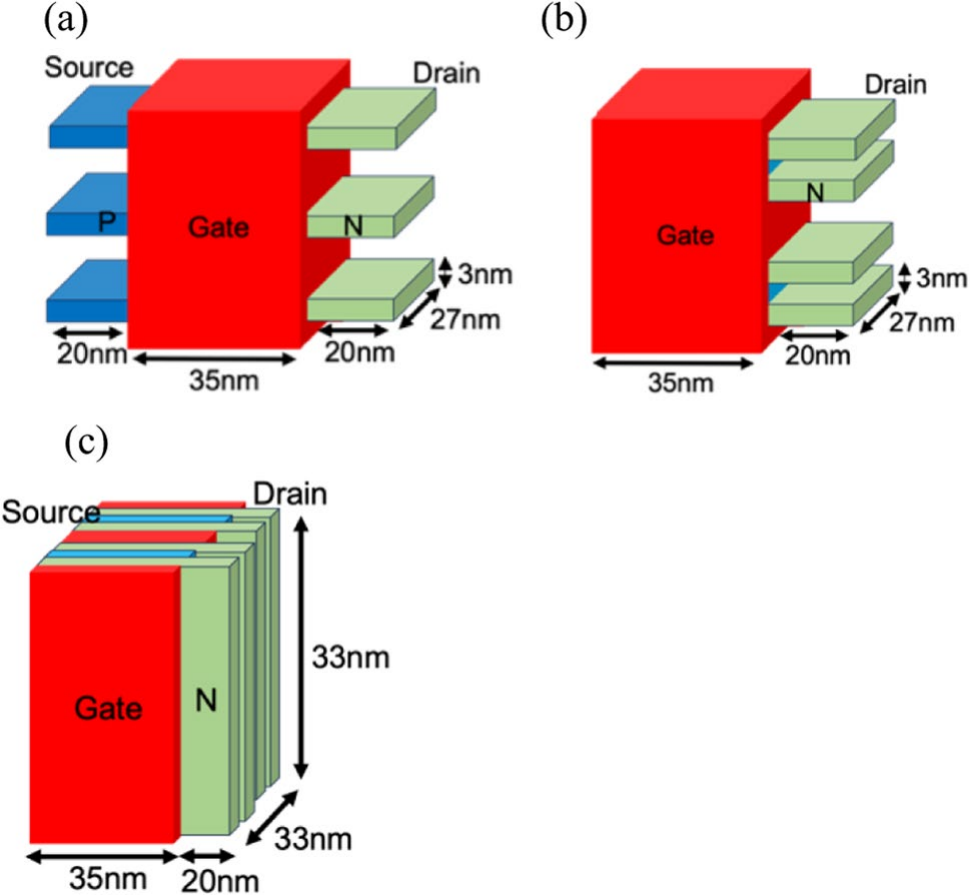
SiGe Body thickness is 3 nm of **a** Nanosheet PIN TFET. **b** Nanosheet iTFET. **c** Fin iTFET

We conducted a comparison of Nanosheet PIN TFET, Nanosheet iTFET, and Fin iTFET with body thicknesses of 5 nm and 3 nm. Figure 1 shows the SiGe Body thickness of 5 nm for Nanosheet PIN TFET, Nanosheet iTFET, and FinFET iTFET. In Fig. 2, the SiGe Body thickness is depicted as 3 nm.

**Fig. 3:**
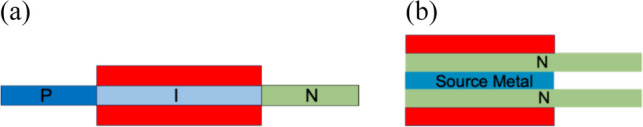
This is a cross-sectional diagram of **a** Nanosheet PIN TFET. **b** Nanosheet iTFET

Figure 3 shows the cross-sectional diagrams of the Nanosheet PIN TFET and Nanosheet iTFET. Various device parameters are presented in Table 1. By replacing traditional Si with SiGe, having a carrier mobility three times that of Si, we achieved superior Band-to-Band characteristics [16]. A PN junction can be formed by utilizing the Schottky contact characteristics of a metal–semiconductor contact with different work functions. To enhance the reliability of the comparison, we employed a uniform overall structure with dimensions of 55 × 25 × 55 (nm^3^) and 55 × 33 × 33 (nm^3^) for length, width, and height, respectively. Although the gate channel length of Nanosheet PIN TFET is 35 nm, the same as the other two, the presence of additional doping regions increased its length by 20 nm, resulting in overall dimensions of 75 × 25 × 55 (nm^3^) and 75 × 33 × 33 (nm^3^). This is a drawback of Nanosheet PIN TFET, requiring a larger volume and incurring higher process costs.

**Table 1:** The SiGe Body thickness is 5 nm/3 nm for device parameters

Parameter	Value
Body thickness (*t*_Body_)	5 nm/3 nm
Gate oxide thickness (*t*_ox_)	5 nm/3 nm
Gate channel length (*L*_G_)	35 nm
Total length of the nanosheet PIN TFET (*L*)	75 nm
Total width of the nanosheet PIN TFET (*W*)	25 nm/33 nm
Total height of the nanosheet PIN TFET (*H*)	55 nm/33 nm
Total length of nanosheet iTFET and Fin iTFET (*L*)	55 nm
Total width of nanosheet iTFET and Fin iTFET (*W*)	25 nm/33 nm
Total height of nanosheet iTFET and Fin iTFET (*H*)	55 nm/33 nm
Source doping concentration in nanosheet PIN TFET (N_A_)	1 × 10^20^ cm^−3^
Channel doping concentration in nanosheet PIN TFET (N_A_)	1 × 10^16^ cm^−3^
Drain doping concentration in nanosheet PIN TFET (N_D_)	1 × 10^18^ cm^−3^
Body doping in nanosheet iTFET (N_D_)	1 × 10^18^ cm^−3^
Body doping in fin iTFET (N_D_)	1 × 10^18^ cm^−3^
Schottky barrier hight (φb)	0.9 eV

Even with efforts to use the same lengths for reliable device comparison, unavoidable adjustments may arise, such as when the body thickness is adjusted from 5 to 3 nm. Considering process-related factors, the gate oxide thickness (*t*_ox_) must also be adjusted with the change in body thickness. Furthermore, in Nanosheet iTFET and Fin iTFET, we employed a uniform doping concentration. In contrast, Nanosheet PIN TFET requires different doping concentrations at various locations to achieve the desired device characteristics. When selecting doping concentrations, we specifically considered the optimal characteristics for each device. Specifically, the doping concentration for Nanosheet iTFET and Fin iTFET is uniformly set at 1 × 10^18^ cm^−3^. Meanwhile, for Nanosheet PIN TFET, the P-type doping concentration is 1 × 10^20^ cm^−3^, the I-type doping concentration is 1 × 10^16^ cm^−3^, and the N-type doping concentration is 1 × 10^18^ cm^−3^. In Sect. 3 we will provide a more detailed description of the optimization of device parameters.

**Fig. 4:**
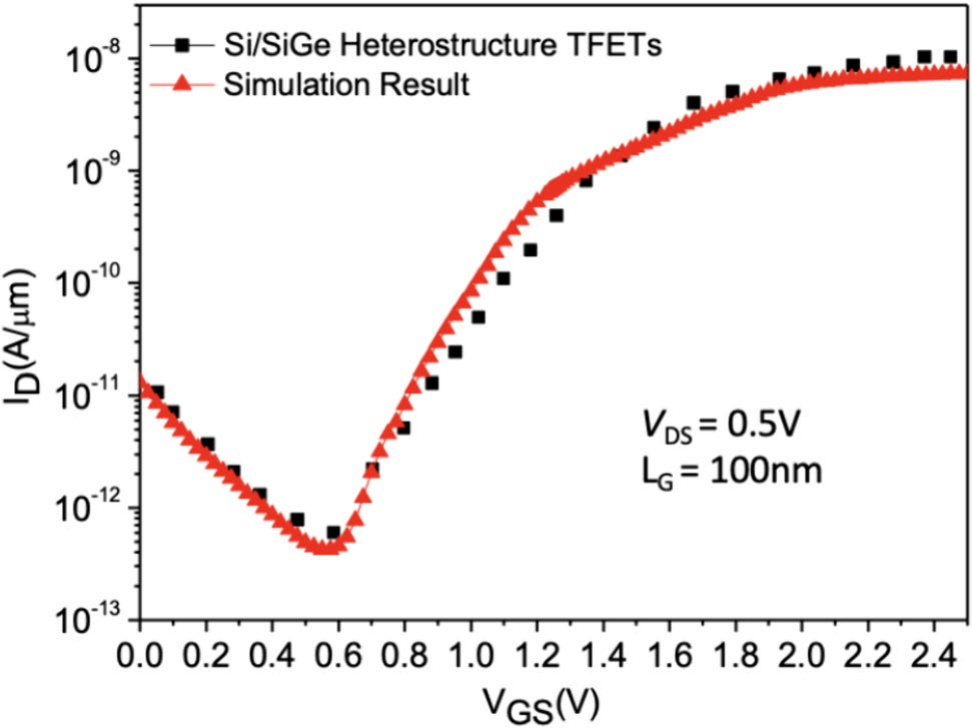
TCAD model calibration using experimental data [17]

In this paper, we employed Sentaurus TCAD to simulate the electrical characteristics of three different types of Tunnel Field-Effect Transistor (TFET) structures proposed by us under various parameter variations. To accurately calculate tunneling currents, we adopted the Dynamic Nonlocal Path Band-to-Band Tunneling Model. Additionally, we accounted for device non-ideal effects, incorporating the Shockley–Read–Hall recombination (SRH) model, Bandgap narrowing, High-field saturation mobility models, Auger recombination model, and considering minute fabrication details, we introduced quantum confinement effects. To ensure the accuracy and feasibility of the simulations, we utilized experimentally fabricated Si/SiGe heterojunctions, considering TFETs that exhibit both line and point tunneling simultaneously [5]. Model calibration for the simulations is illustrated in Fig. 4.

**Fig. 5:**
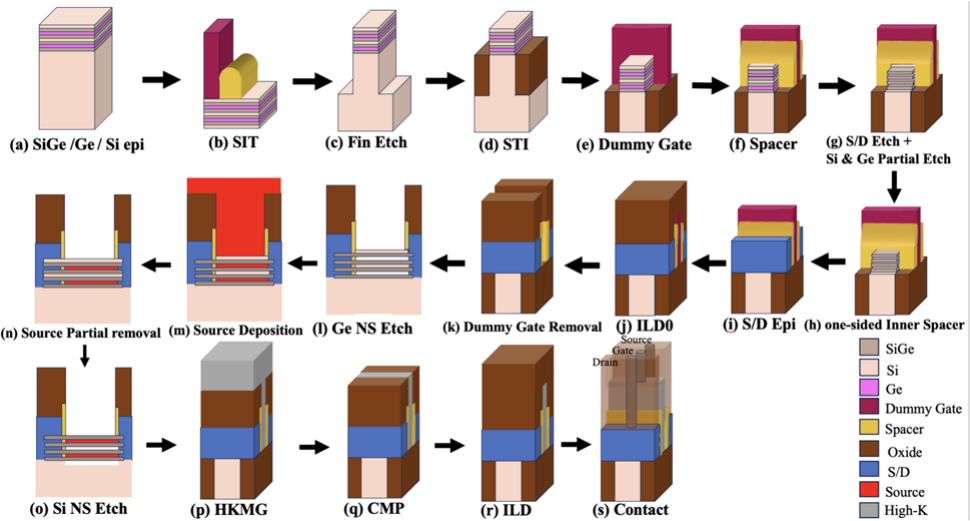
**a**–**s** Fabrication process steps of stacked SiGe Nanosheet iTFET

The fabrication process for the stacked SiGe nanosheet iTFET is shown in Fig. 5. To begin, multi-layer SiGe/Si/Ge stacks, each with 5 nm Ge_0.3_Si_0.7_ and 5 nm Si, were grown in a reduced-pressure chemical vapor deposition (RPVVD) chamber in Fig. 5a. Following the stack growth, fin arrays patterns were precisely created utilizing the spacer image transfer (SIT) technique, achieving a resolution beyond that of advanced photolithography. This meticulous method guaranteed the accurate definition of the intended fin structures in Fig. 5b. To define the fins, fin etching was performed, shaping, refining the structures to the required specifications in Fig. 5c. Shallow trench isolation (STI) was introduced, while a SiO_2_ with a high aspect ratio process (HARP) was deposited to enhance the overall structure, providing essential isolation for subsequent transistor components. To reveal the fin, diluted hydrofluoric acid (DHF) was used to perform a SiO_2_ etching process in Fig. 5d. A dummy gate stack was formed on the fins during the execution of dummy gate formation in Fig. 5(e). SiO_2_ spacers were carefully formed in Fig. 5f. S/D cavity etching and partial etching of Ge and Si were performed in Fig. 5g. A one-sided inner spacer was meticulously achieved by depositing a thin layer of SiNx and employing the Reactive Ion Etching (RIE) process in Fig. 5h. The Ge_0.3_Si_0.7_ epitaxy process with in-situ doping was followed by the epitaxial growth of the source and drain regions in Fig. 5i. ILD0 deposition was carried out in Fig. 5j. Dummy gate was promptly eliminated through immersion in tetramethylammonium hydroxide in Fig. 5k. The release of Ge Nanosheet (NS) channels through selective etching in Fig. 5l. Source metal deposition in Fig. 5m. Source metal partially removed in Fig. 5n. Selective etching released Si Nanosheet (NS) channels in Fig. 5o. The multilayer High-K Metal Gate (HKMG) film stacks were applied using an Atom Layer Deposition (ALD) method. In Fig. 5p. Chemical mechanical planarization (CMP) was applied, smoothing, and refining the device’s surface in Fig. 5q. ILD deposition was carried out in Fig. 5r. Finally, metal deposition and contact were established in Fig. 5s [18–21].

**Fig. 6:**
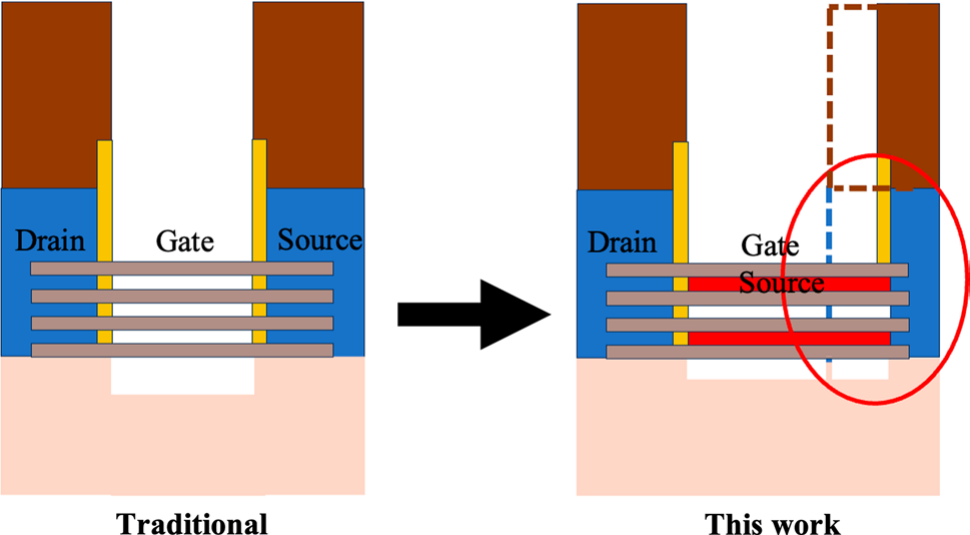
Our devices exhibit a larger overlap area between the Gate and Source metals compared to traditional components, within the same volume

Compared to traditional devices, Nanosheet iTFET exhibits a larger overlap area between the gate and source metal within the same volume, as illustrated in Fig. 6. This is advantageous for the iTFET, which relies primarily on the line tunneling mechanism, where tunneling current is proportional to the width (*W*) and length (*L*). Therefore, increasing the overlap area allows the iTFET to demonstrate excellent circuit characteristics [22, 23].

## Electrical characteristics discussion

### Comparative analysis of three semiconductor devices and the benefits of stacking

**Fig. 7:**
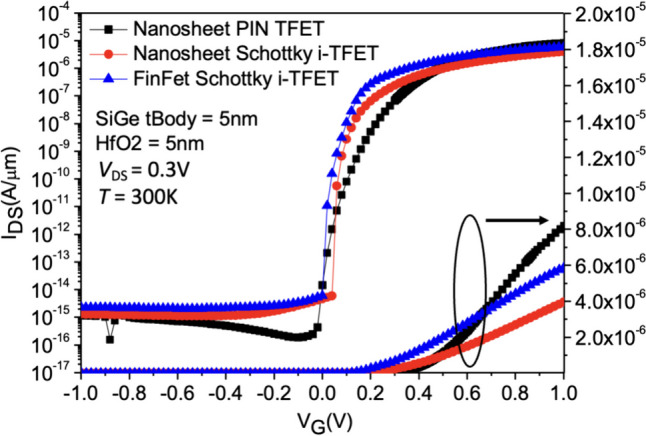
*I*_D_–*V*_G_ characteristic curves of the three devices with both SiGe Body thickness and gate oxide thickness set to 5 nm

**Fig. 8:**
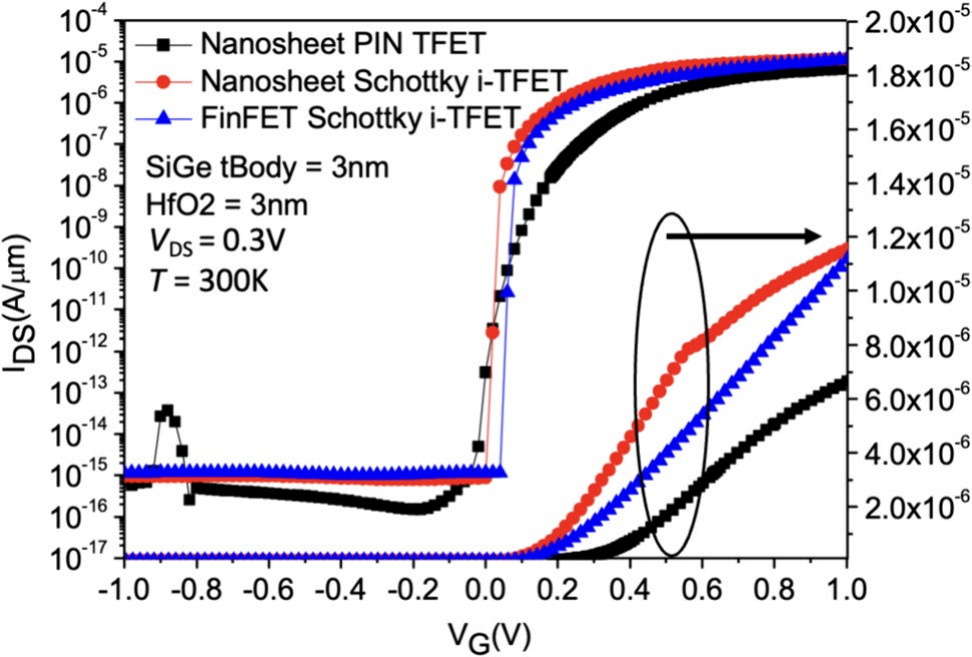
*I*_D_–*V*_G_ characteristic curves of the three devices with both SiGe Body thickness and gate oxide thickness set to 3 nm

Figures 7 and 8 show the *I*_D_–*V*_G_ characteristics of Nanosheet PIN TFET, Nanosheet iTFET, and Fin iTFET, respectively. When the SiGe Body thickness is 5 nm, the Nanosheet PIN TFET, utilizing the traditional point tunneling mechanism, exhibits a significantly higher *I*_ON_ compared to the other two; however, this is accompanied by a larger subthreshold swing (*SS*). To address this, we further explore Nanosheet iTFET and Fin iTFET, which primarily utilize the line tunneling mechanism, to achieve a smaller *SS*.

Nevertheless, the *I*_ON_ of these two devices is relatively lower. When the SiGe Body thickness is reduced to 3 nm, Nanosheet iTFET and Fin iTFET, benefitting from a thinner substrate, showcase a slight increase in *I*_ON_ due to enhanced tunneling capabilities. This improvement successfully mitigates the issue of lower *I*_ON_. In contrast, Nanosheet PIN TFET exhibits a slight decrease in *I*_ON_ and a minor ambipolar effect, leading to an increase in leakage current and a reduction in *I*_ON_/I_OFF_ ratio. Therefore, Nanosheet iTFET and Fin iTFET perform better at a SiGe Body thickness of 3 nm, making them more ideal choices for smaller sizes. Additionally, when the SiGe Body thickness is 5 nm, the *SS*_avg_ for Nanosheet PIN TFET is 47.63 mV/dec, while for Nanosheet iTFET and Fin iTFET, it is 22.48 mV/dec and 20.22 mV/dec, respectively. At a SiGe Body thickness of 3 nm, the *SS* for all three structures shows a slight improvement. A comparative analysis of the three components in Table 2.

**Table 2:** Characteristics comparison of these three devices under different SiGe body thickness conditions

	Nanosheet PIN TFET	Nanosheet iTFET	FinFET iTFET
*I*_ON_/*I*_OFF_ (SiGe Body = 5 nm)	7.89 $\times$ 10^8^	1.21 $\times$ 10^8^	2.1 $\times$ 10^8^
*SS*_avg_ (mV/dec) (SiGe Body = 5 nm)	47.63	22.48	20.22
Length × Width × Height (SiGe Body = 5 nm)	75 $\times$ 25 $\times$ 55 nm^3^	55 $\times$ 25 $\times$ 55 nm^3^	55 $\times$ 25 $\times$ 55 nm^3^
*I*_ON_/*I*_OFF_ (SiGe Body = 3 nm)	1.2 $\times$ 10^8^	5.29 $\times$ 10^9^	2.7 $\times$ 10^9^
*SS*_*avg*_ (mV/dec) (SiGe Body = 3 nm)	29.19	17.64	17.48
Length × Width × Height (SiGe Body = 3 nm)	75 $\times$ 33 $\times$ 33 nm^3^	55 $\times$ 33 $\times$ 33 nm^3^	55 $\times$ 33 $\times$ 33 nm^3^
Cost	High (Implantation doping)	Low	Low

**Fig. 9:**
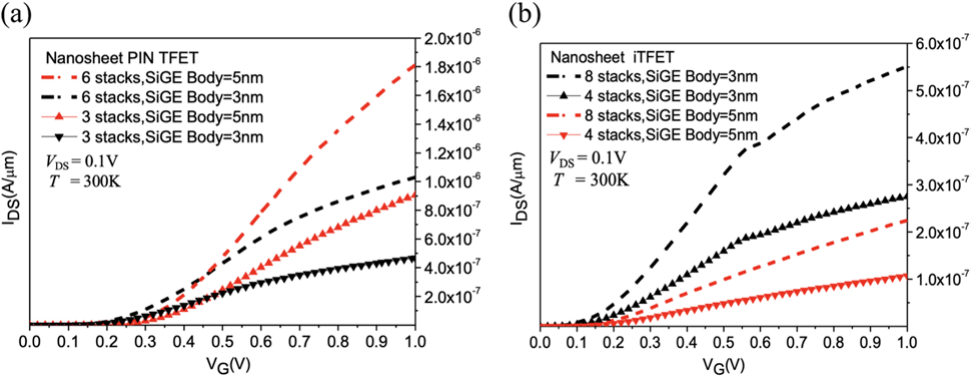
**a** *I*_D_–*V*_G_ characteristic curves of the PIN TFET, **b** of the Nanosheet iTFET, with varying SiGe Body thicknesses, as a function of increasing stack layers

Figure 9 shows the current variation plots for Nanosheet PIN TFET and Nanosheet iTFET at different SiGe Body thicknesses and varying the number of sheets. It is observed that with an increase the number of sheets, both Nanosheet PIN TFET and Nanosheet iTFET exhibit a rising trend in *I*_ON_ current. Thus, by employing stacking, we effectively enhance the *I*_ON_ performance of TFET, addressing the relatively lower *I*_ON_ scenario while achieving lower *SS* and reduced power consumption.

**Fig. 10:**
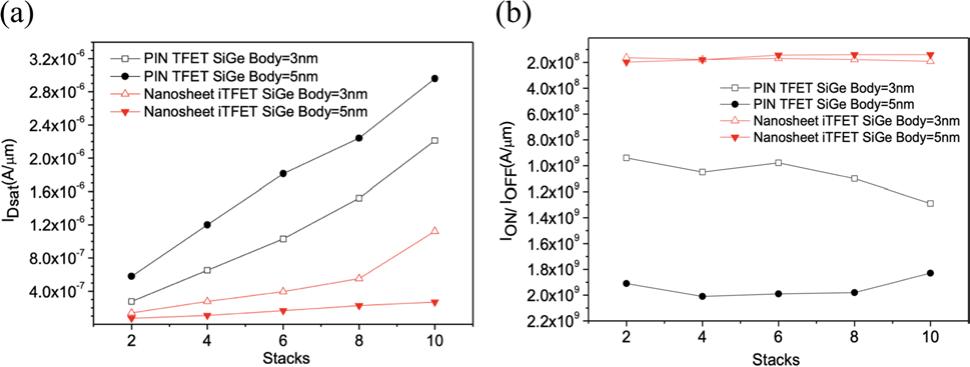
**a** The total on current (*I*_Dsat_), **b** I_ON/_I_OFF_versus the number of sheets. In the on-state, the current variation changes with an increase in the number of stack layers

**Fig. 11:**
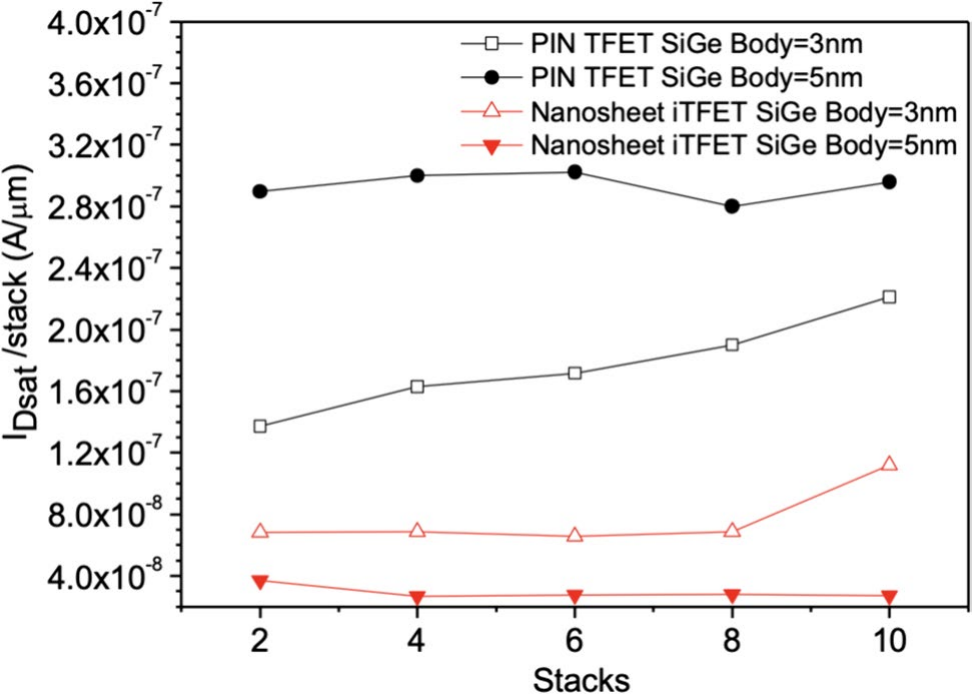
The total on current (*I*_Dsat_) per average layer versus the number of sheets. In the on-state, the current efficiency brought by each layer’s stack number

Upon further increasing the number of sheets, we conducted observations on Nanosheet PIN TFET and Nanosheet iTFET in Fig. 10a. Increasing the number of sheets indeed leads to an increase in *I*_ON_, but it also comes with an increase in *I*_OFF_. However, it’s important to consider the overall change in *I*_ON_/*I*_OFF_ ratio. Even if both *I*_ON_ and *I*_OFF_ increase, if their increase is relatively small, the overall change in *I*_ON_/*I*_OFF_ ratio won’t have a significant impact on device performance. Therefore, increasing the number of sheets can effectively address the issue of low *I*_ON_ in TFETs while maintaining a good *I*_ON_/*I*_OFF_ ratio, as depicted in Fig. 10b. We calculated the average *I*_ON_ gain per stack for each component, revealing that although the *I*_ON_ gain per stack does not linearly increase with the number of stacks, it also does not decrease in Fig. 11. This allows us to effectively improve *I*_ON_ while increasing the number of sheets, without concern for potential adverse effects on the device.

**Fig. 12:**
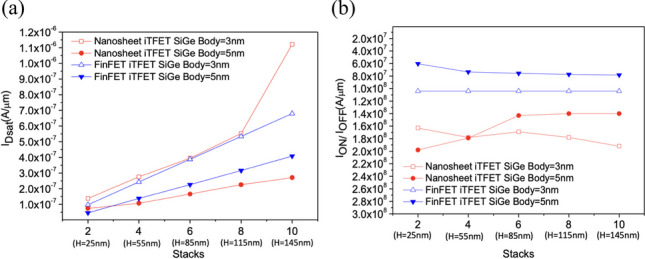
**a** The total on current (*I*_Dsat_), **b** *I*_ON/_*I*_OFF_ versus the number of sheets. In the on-state, with both components having the same volume, the current variation will change as the number of stacked layers increases

**Fig. 13:**
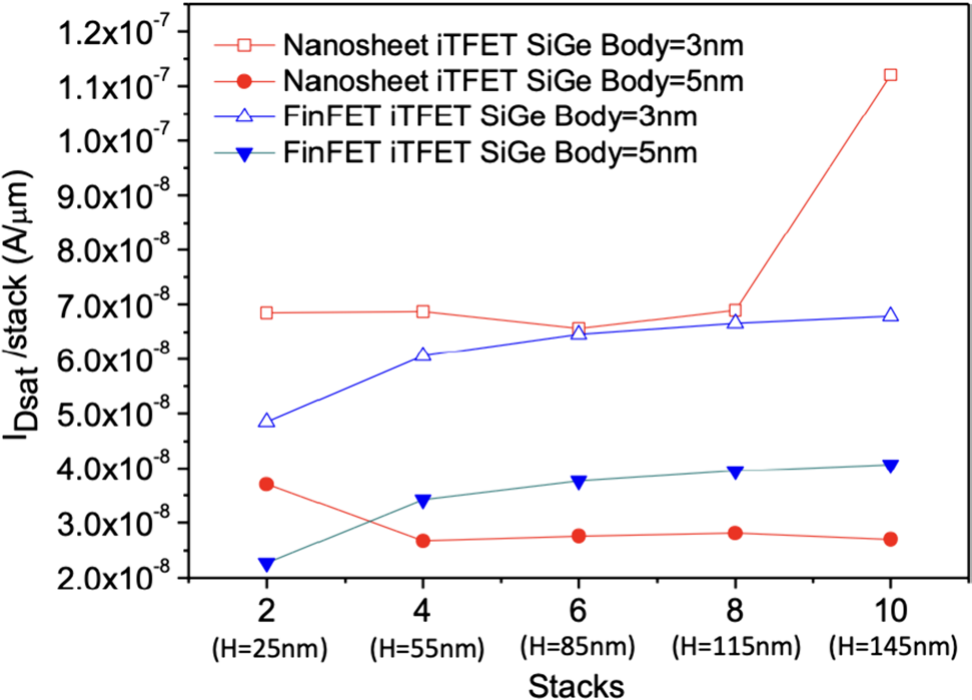
The total on current (*I*_Dsat_) per average layer versus the number of sheets. In the on-state, with both components having the same volume, the current efficiency brought by each layer’s stack number

Figure 12a shows the current variation of Nanosheet iTFET and Fin iTFET with different numbers of stacked layers under the same volume. For example, with SiGe Body = 5 nm, in the case of 2 stacked layers, the dimensions are $55\times 25\times 25$ (nm^3^), and for 4 stacked layers, the dimensions are $55\times 25\times 55$ (nm^3^). In this comparable scenario, we examined whether the benefits of stacking surpass those of Fin iTFET. The results indicate that for SiGe Body = 5 nm, the average benefits of Fin iTFET are greater than those of Nanosheet iTFET. Figure 12b also indicates that there won’t be significant changes in *I*_ON_/*I*_OFF_. However, for SiGe Body = 3 nm, Nanosheet iTFET exhibits better average benefits in Fig. 13. This is because Nanosheet iTFET already performs optimally among these three components at SiGe Body = 3 nm.

### Optimization and analysis of device parameters

**Fig. 14:**
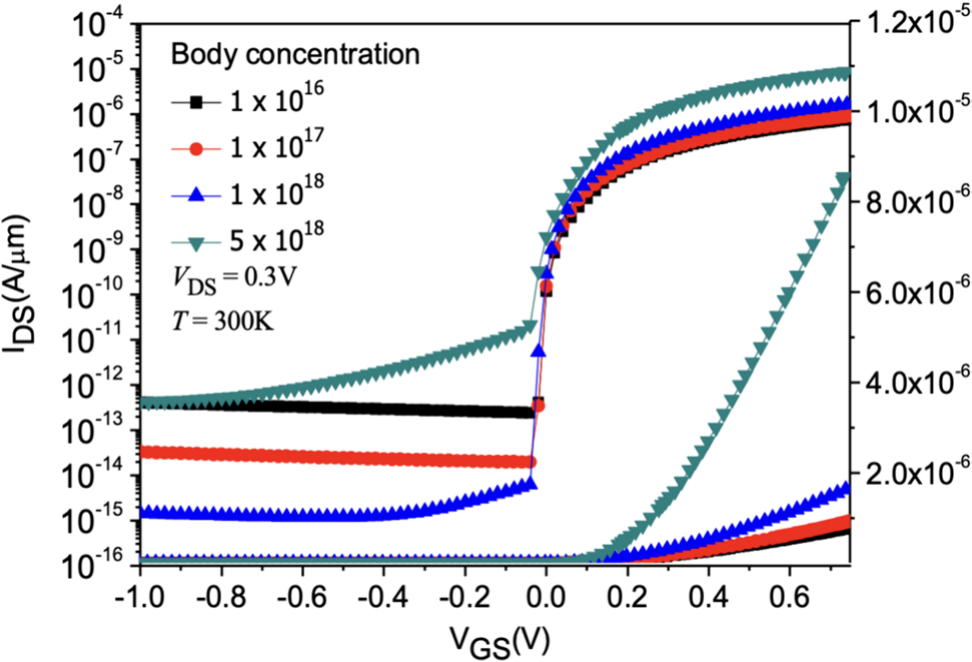
*I*_D_–*V*_G_ characteristic curves for different SiGe body concentrations of the Nanosheet iTFET

**Fig. 15:**
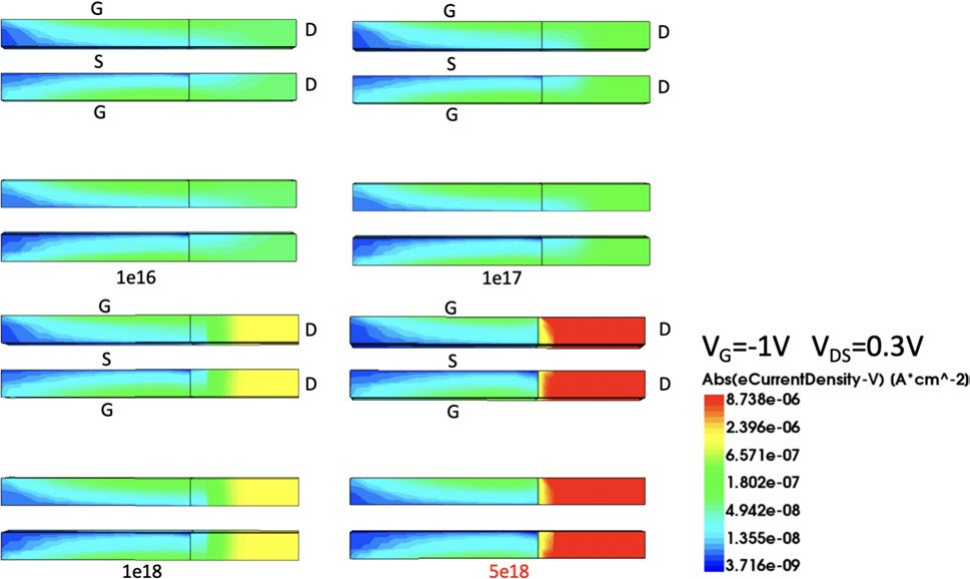
Electron current density in the off-state for different SiGe body concentrations of the Nanosheet iTFET

**Fig. 16:**
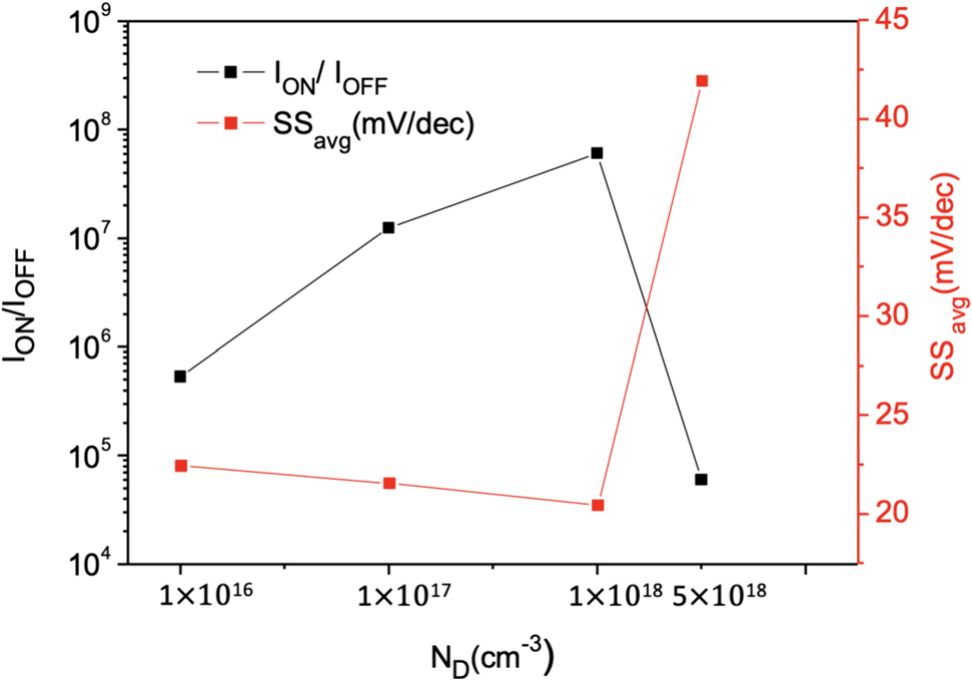
*I*_ON_/*I*_OFF_, *SS*_avg_, for different SiGe body concentrations of the Nanosheet iTFET

Figure 14 shows the current variation plot of Nanosheet iTFET at different SiGe Body concentrations. Due to the operation of TFET being based on quantum tunneling effects, electrons tunnel through the band bending. We observe that with the increase in SiGe Body concentration, the height of the barrier changes, allowing more electrons to easily tunnel across the barrier. This results in the device achieving higher *I*_ON_ in the on-state. In the off-state, as the concentration increases, the device exhibits superior control, achieving lower *I*_OFF_ and consequently reducing the overall *SS*. However, when the concentration becomes too high, despite the increase in *I*_ON_ in the on-state, it also generates a significant amount of carrier tunneling, markedly increasing the leakage current, as shown in Fig. 15. Therefore, we choose $1\times {10}^{18}$ cm^−3^ as the substrate concentration for the device, as it performs better in terms of *I*_ON_/*I*_OFF_ and *SS*_avg_, as detailed in Fig. 16.

**Fig. 17:**
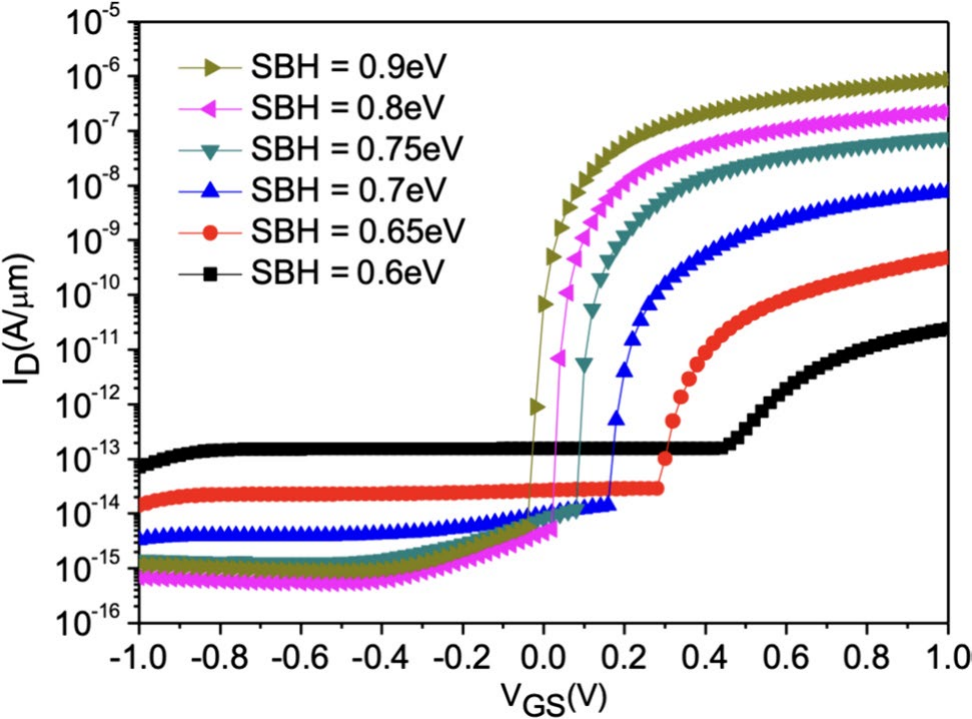
*I*_D_–*V*_G_ characteristic curves for different Schottky barrier heights of the Nanosheet iTFET

**Fig. 18:**
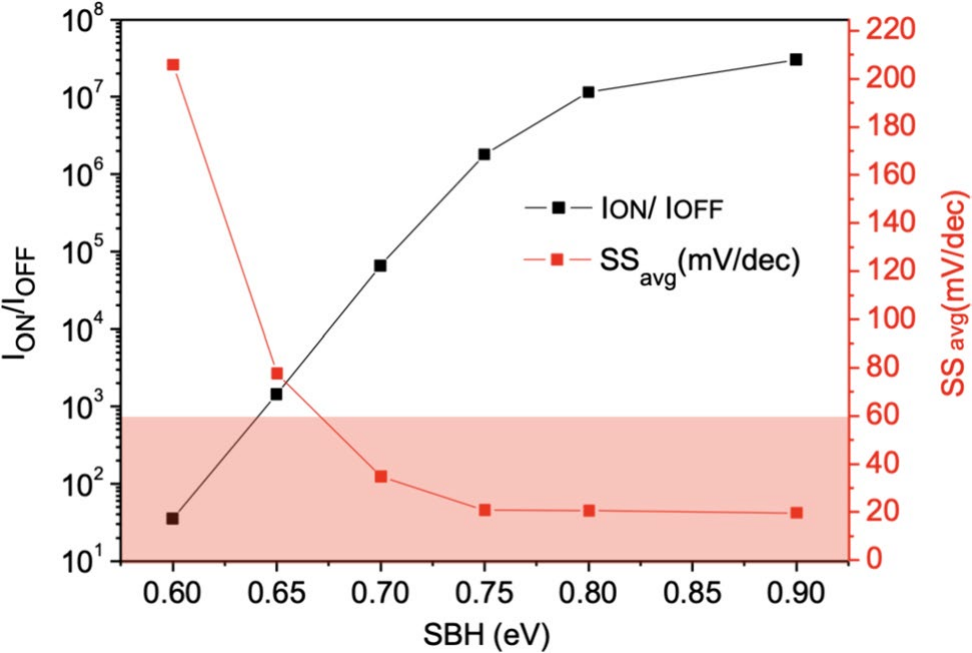
*I*_ON_/*I*_OFF_, *SS*_avg_, for different Schottky barrier heights of the Nanosheet iTFET

**Fig. 19:**
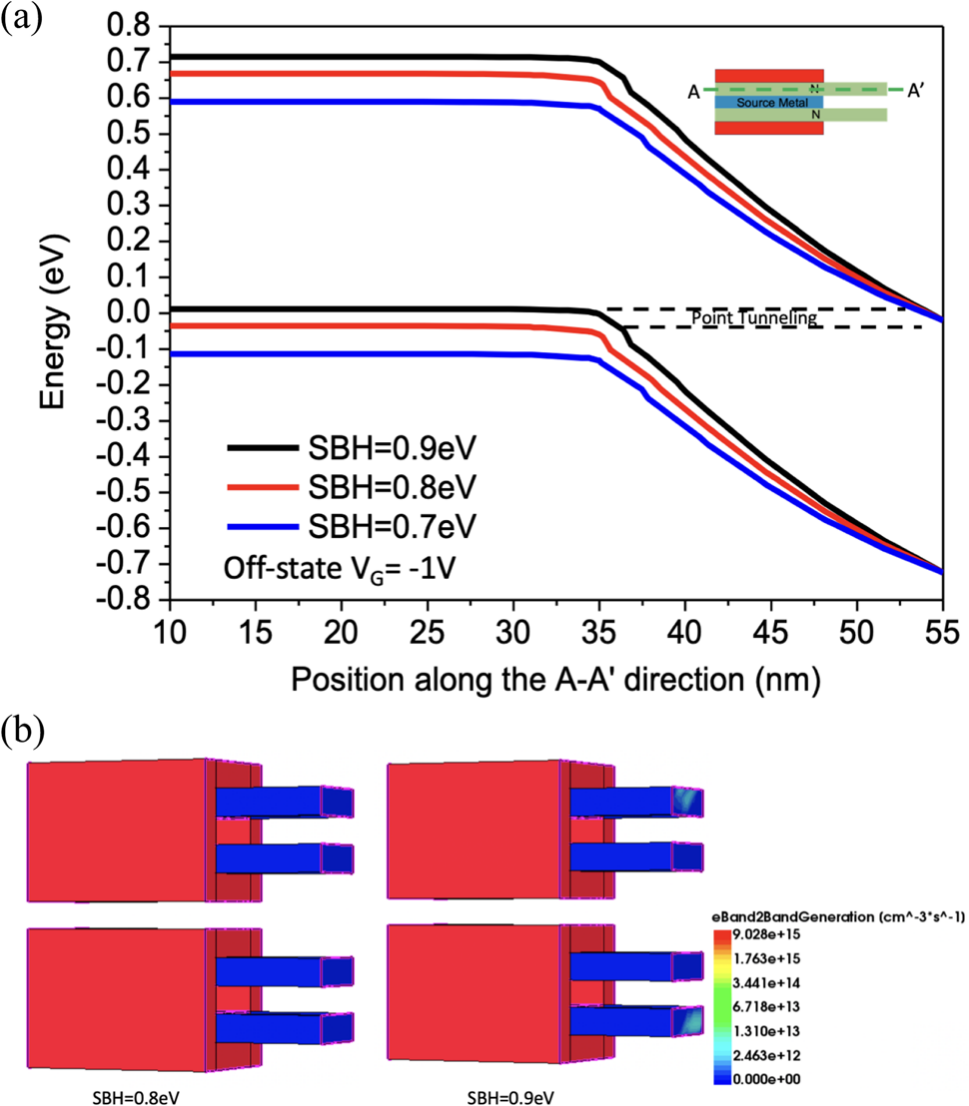
**a** Band diagram, **b** Band-to-band generation for different Schottky barrier heights of the Nanosheet iTFET

Figure 17 shows the current variation with different Schottky barrier heights (φb). To achieve Schottky contact, we observe that when φb < 0.7 eV, thermal electron injection is prominent. Conversely, when φb > 0.7 eV, the dominant mechanism in the device gradually transitions from thermal electron injection to tunneling, allowing *SS* to decrease below 60 mV/dec, as shown in Fig. 18. With the increase in φb, both *I*_ON_ and *SS* exhibit superior performance [24]. When the band bending reaches a certain degree, leakage current also starts to increase due to tunneling in the off state. It can be observed that when φb = 0.9, the tunneling effect on leakage current becomes more pronounced, resulting in a slightly higher *I*_OFF_ compared to φb = 0.8. This suggests that an increase in φb does not necessarily lead to an absolute improvement in device performance in Fig. 19a, b.

**Fig. 20:**
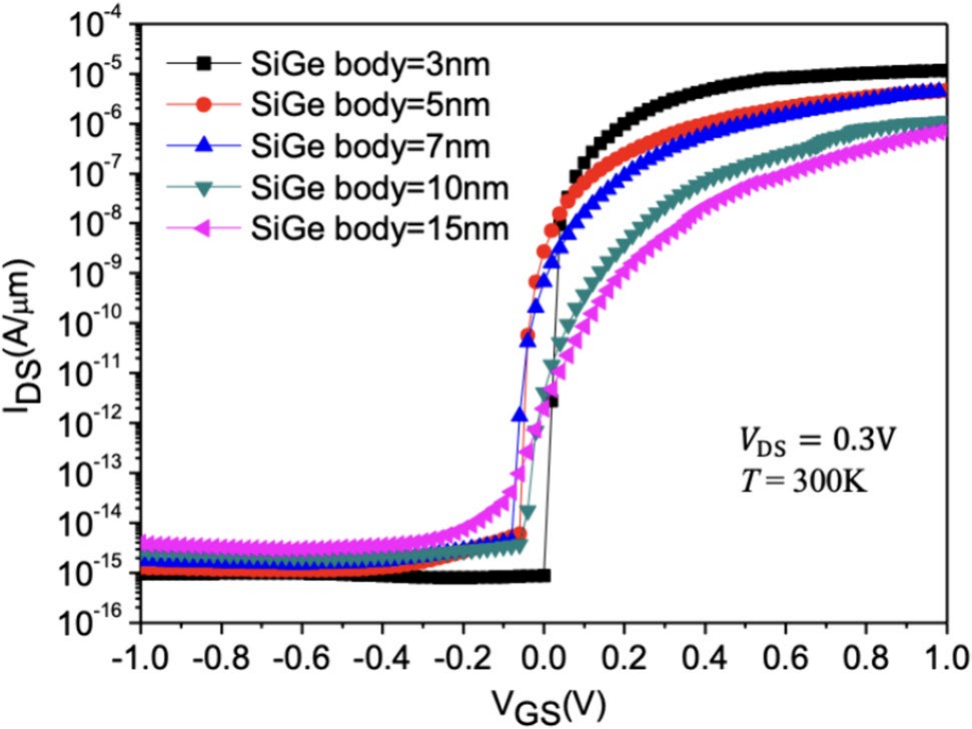
*I*_D_–*V*_G_ characteristic curves for different SiGe body thickness of the Nanosheet iTFET

**Fig. 21:**
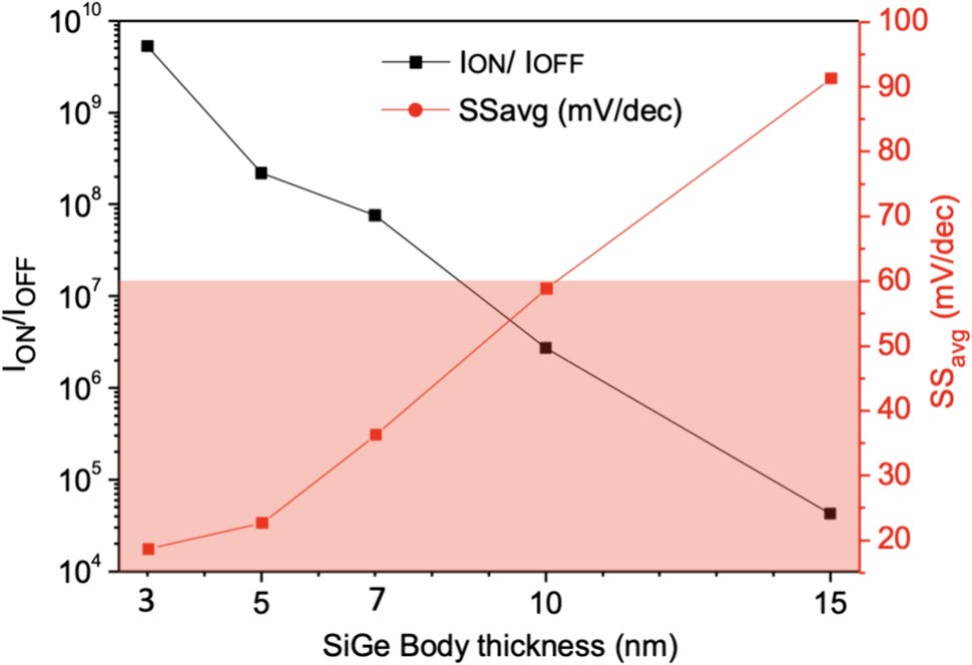
*I*_ON_/*I*_OFF_, *SS*_avg_, for different SiGe body thickness of the Nanosheet iTFET

Figure 20 shows the current variation at different SiGe Body thicknesses. As the Nanosheet iTFET primarily adopts the line tunneling mechanism, a thinner substrate not only reduces the device’s volume but also shortens the tunneling distance between the Gate and Source, enhancing the Band-to-Band tunneling effect and thus improving device performance. Therefore, the choice of substrate thickness is crucial for TFET performance. We observe that when the SiGe Body thickness is 3 nm, it not only exhibits optimal *I*_ON_/*I*_OFF_ but also achieves the lowest *SS* and minimal volume. Conversely, when the SiGe Body thickness > 10 nm, the tunneling effect becomes less favorable, leading to an *SS* exceeding 60 mV/dec, which compromises the TFET’s advantage in rapid switching compared to MOSFET in Fig. 21.

### The non-ideal effects of the device and improvement

**Fig. 22:**
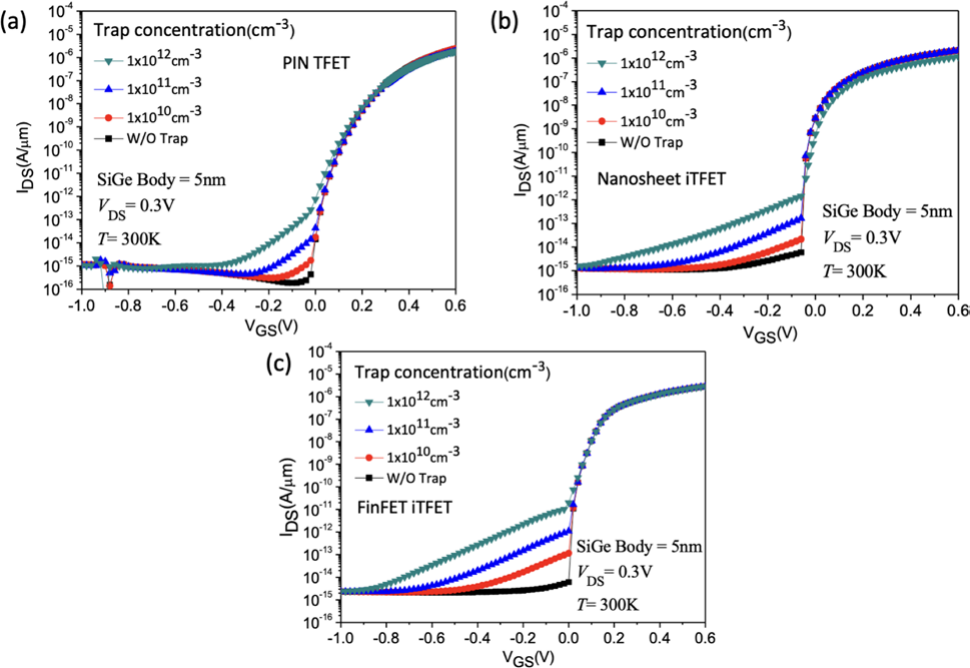
**a** *I*_D_–*V*_G_ characteristic curves of the PIN TFET, **b** of the Nanosheet iTFET, **c** of the Fin iTFET for different interface trap densities

The presence of interface traps has been observed to reduce the conduction current in tunneling TFETs, primarily relying on the tunneling interface lateral electric field peak. Since interface traps are only present on the interface at channel-drain tunneling junction only, the on-current is likely unaffected by interface traps. However, the ambipolar conduction induced by the motion of charged carriers at the output tunneling interface is significantly influenced by interface traps. It can be noted that both points tunneling-dominated PIN TFET and line tunneling-dominated Nanosheet iTFET are affected by traps, as illustrated in Fig. 22a–c. To mitigate the impact of traps on device performance, apart from employing High-K materials proposed by others in this device, we have suggested several methods to ameliorate the effects of traps [25–27].

**Fig. 23:**
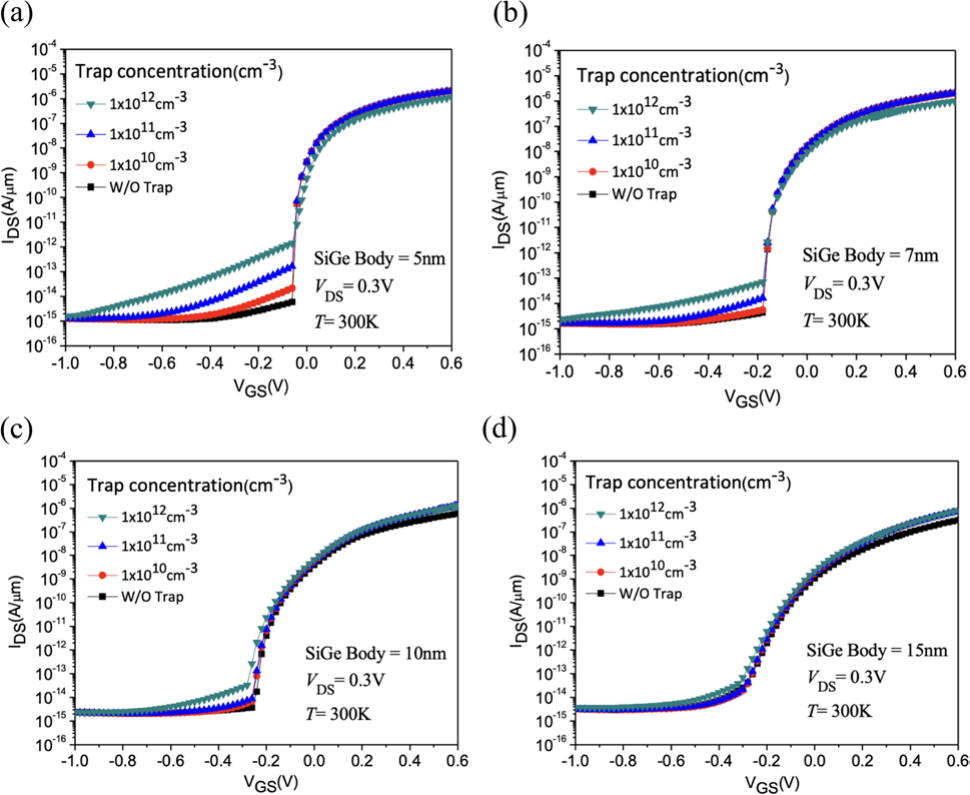
*I*_D_–*V*_G_ characteristic curves with SiGe Body thicknesses of **a** 5nm, **b** 7nm, **c** 10nm, **d** 15nm, as a function of different interface trap densities of the Nanosheet iTFET

Since interface traps typically occur at the interface between HfO2 and SiGe Body, with the increase in interface trap density, the negative charge density at the interface increases, thereby increasing the electron concentration at the interface. This situation induces band bending in the interface tunneling oxide. The introduction of traps creates new tunneling channels for charge carriers to cross the bandgap, resulting in leakage current occurring even in the off state due to tunneling. Therefore, the occurrence of tunneling phenomena leads to an increasing trend in the leakage current profile. We first focus on the discussion of SiGe Body [28, 29]. We varied the SiGe Body thickness of the Nanosheet iTFET from 5 to 15 nm. The results show that at a SiGe Body thickness of 5 nm, interface traps have a noticeable impact on the device. However, as the SiGe Body thickness increases, the influence of traps gradually diminishes, especially when SiGe Body = 15 nm, the device is almost entirely unaffected by traps, as shown in Fig. 23a–d. It’s essential to note that since our device is primarily line tunneling-dominated, increasing the SiGe Body thickness reduces the device’s line tunneling control capability. Besides increasing the device volume, the subthreshold swing also increases accordingly. As mentioned earlier, when SiGe Body > 10 nm, the SS exceeds 60 mV/dec. Considering these results, increasing the SiGe Body thickness is not an effective way to mitigate the impact of traps.

**Fig. 24:**
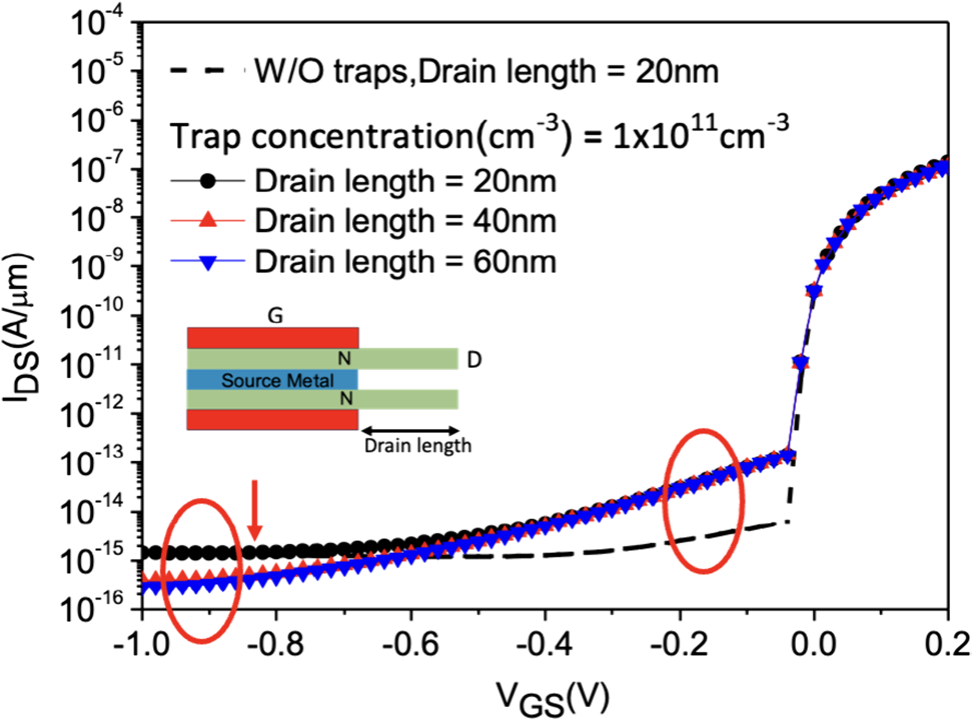
*I*_D_–*V*_G_ characteristic curves with different Drain length of the Nanosheet iTFET

While increasing the drain length to lengthen the distance between the gate and drain can effectively reduce* I*_OFF_, which is a well-known technique [30], it becomes evident that adjusting the drain length is not an effective means to mitigate the impact of traps, especially when considering the presence of interface traps. This is illustrated in Fig. 24. Clearly, the adjustment of drain length is not an effective approach to address the influence of traps.

**Fig. 25:**
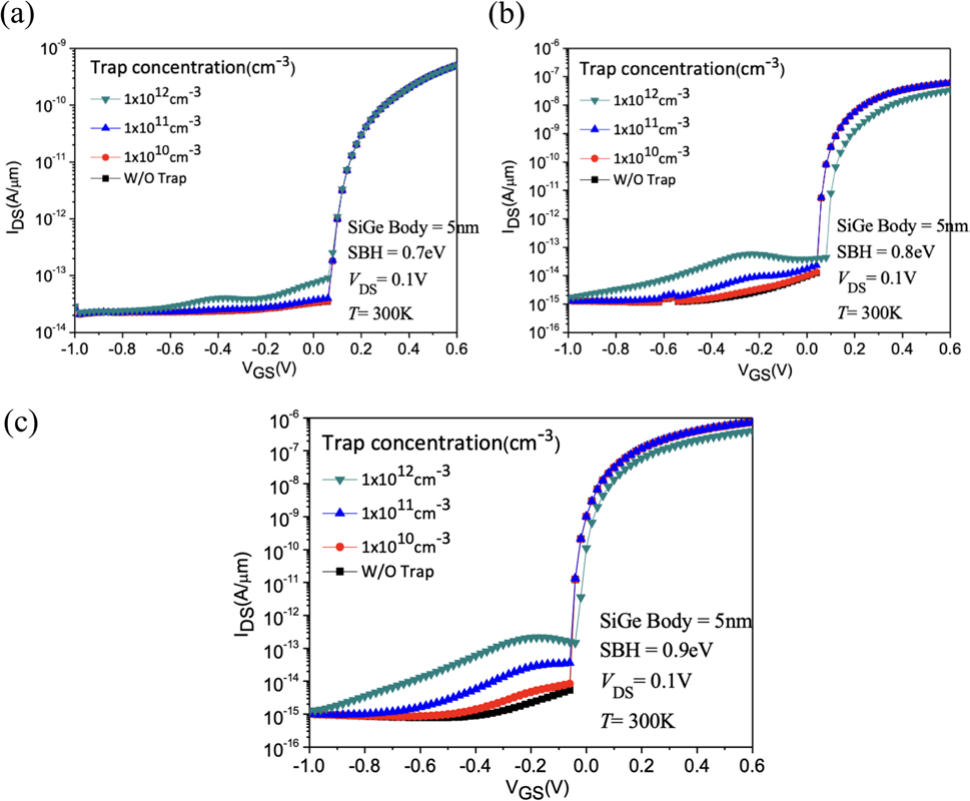
*I*_D_–*V*_G_ characteristic curves with Schottky barrier heights of **a** 0.7 eV, **b** 0.8 eV, **c** 0.9 eV, as a function of diverse interface trap densities of the Nanosheet iTFET

By altering the source metal, we indirectly adjusted the Schottky barrier height (*φ*_b_) [17]. At (*φ*_b_) = 0.9 eV, the device achieves better* I*_ON_/*I*_OFF_ and lower *SS*. However, when considering the non-ideal effects of interface traps, increasing (*φ*_b_) not only effectively enhances *I*_ON_/*I*_OFF_ but also has a significant impact on traps in Fig. 25a–c. Therefore, adjusting (*φ*_b_) resulted in substantial trap influence at (*φ*_b_) = 0.9 eV, and almost complete immunity to traps at (*φ*_b_) = 0.7 eV. However, at (*φ*_b_) = 0.7 eV, the tunneling-dominant mechanism is not ideal. Consequently, we consider (*φ*_b_) = 0.8 eV to be the most suitable choice for the device. This option minimizes the impact of traps while preserving favorable tunneling characteristics.

**Fig. 26:**
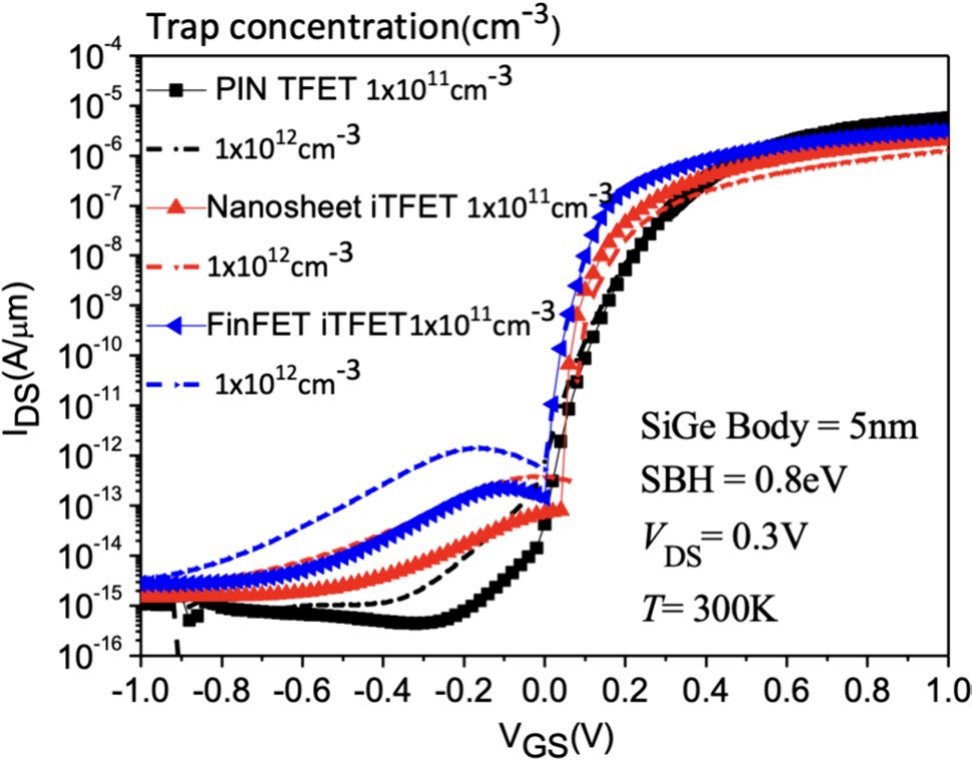
*I*_D_–*V*_G_ characteristic curves of the three devices with different interface trap densities at Schottky barrier height = 0.8 eV

**Fig. 27:**
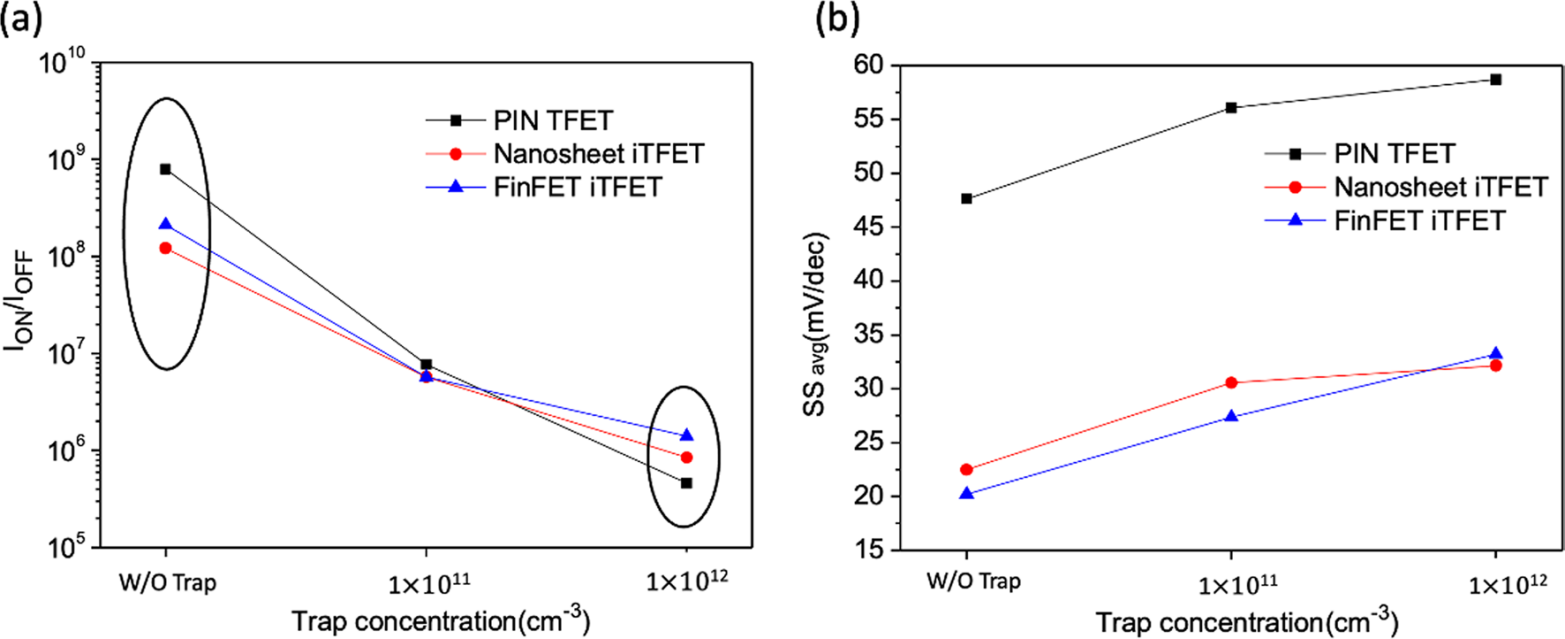
**a** I_ON_/I_OFF_ ratio of the three devices **b** SS with different interface trap densities at Schottky barrier height = 0.8 eV

We applied (*φ*_b_) = 0.8 eV to all three devices and conducted a device analysis under different interface trap conditions in Fig. 26. The results indicate that, without considering interface traps, the PIN TFET using the point tunneling mechanism exhibits superior *I*_ON_/*I*_OFF_. However, with the increase in interface trap concentration, Nanosheet iTFET and Fin iTFET, utilizing the line tunneling mechanism, outperform in terms of *I*_ON_/*I*_OFF,_ as shown in Fig. 27a. Regarding subthreshold swing (*SS*), all three devices show an increase as the interface trap concentration rises. Even at a relatively high interface trap concentration of 1 $\times 10$^12^, PIN TFET maintains an *SS* below 60 mV/dec, while Nanosheet iTFET and Fin iTFET demonstrate commendable performance with an *SS* of 33 mV/dec in Fig. 27b.

**Fig. 28:**
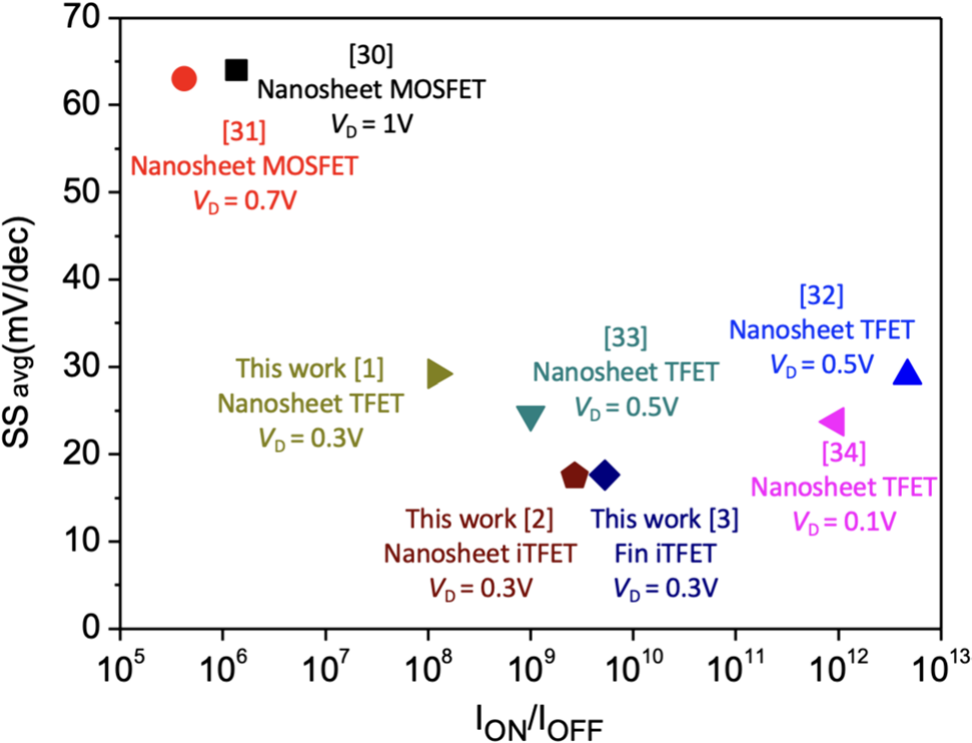
Performance comparison of Stacked Nanosheet semiconductor devices

By comparing our device with stacked Nanosheet devices reported in recent years, our device demonstrates a lower subthreshold swing at lower power supply voltages in Table 3 and Fig. 28 [3, 31–34]. While the *I*_ON_/*I*_OFF_ ratio may be slightly lower compared to other reference literature, we believe that users can choose an appropriate number of stacking layers based on their specific *I*_ON_ requirements, thus addressing the challenge of maintaining a low subthreshold swing while improving the issue of too low *I*_ON_/*I*_OFF_.

**Table 3:** Comparison of the proposed Stacked nanosheet semiconductor devices

	Structure	Material	*I*_ON_ (A/μm)	V_D_ (V)	SS_avg_ (mV/dec)	*I*_ON_/*I*_OFF_
Ref. [3]	Nanosheet MOSFET	Si	$3\times {10}^{-3}$	1	64	$1.36\times {10}^{6}$
Ref. [31]	Nanosheet MOSFET	Si	$4\times {10}^{-5}$	0.7	63	$4.2\times {10}^{5}$
Ref. [32]	Nanosheet TFET	Si	$2.1\times {10}^{-4}$	0.5	29	$4.73\times {10}^{12}$
Ref. [33]	Nanosheet TFET	Si	$2\times {10}^{-6}$	0.5	24.6	$1\times {10}^{9}$
Ref. [34]	Nanosheet TFET	Si	$1\times {10}^{-6}$	0.1	23.7	$9.38\times {10}^{11}$
This work 1	Nanosheet TFET	SiGe	$6\times {10}^{-7}$	0.3	29.19	$1.2\times {10}^{8}$
This work 2	Nanosheet iTFET	SiGe	$4.6\times {10}^{-6}$	0.3	17.64	$5.29\times {10}^{9}$
This work 3	Fin iTFET	SiGe	$3.2\times {10}^{-6}$	0.3	17.48	$2.7\times {10}^{9}$

## Conclusion

In this thesis, we employ a line tunneling mechanism and applying Nanosheet stacking method, combined with iTFET technology. This has successfully achieved superior Band-to-Band characteristics, lower subthreshold swing, and higher *I*_ON_. In terms of the fabrication process, using the line tunneling mechanism in iTFET allows Nanosheet iTFET to have a larger gate and source overlap area, compared to traditional Nanosheet MOSFETs of the same volume, thereby enhancing device performance. Within Nanosheet iTFET, we have also investigated SiGe Body thickness and SiGe Body concentration. The results indicate optimal device performance when SiGe Body = 3 nm and SiGe Body concentration is 1 $\times$ 10^18^.

However, under non-ideal conditions, interface traps pose a significant challenge for TFETs. We conducted an in-depth study on interface traps and found that (*φ*_b_) = 0.8 eV is the most suitable choice for the device. This not only effectively reduces the impact of traps but also maintains excellent tunneling characteristics. Additionally, we compared PIN TFET utilizing point tunneling and Nanosheet iTFET and Fin iTFET utilizing line tunneling. Ultimately, we discovered that Nanosheet iTFET or Fin iTFET with line tunneling not only achieves higher *I*_ON_/*I*_OFF_ but also reaches a minimum *SS* of 17 mV/dec. Even considering interface traps, the worst-case scenario for *SS* remains below 33 mV/dec. This comprehensive comparison contributes valuable insights for the design and optimization of stacked semiconductor devices.

In future applications such as IoT and AI devices, the increasing demand for higher voltages has become an unavoidable issue in terms of device power consumption. Maintaining high performance and steep subthreshold swing becomes crucial as the supply voltage decreases. Based on the results above, we believe that Nanosheet iTFET will become the preferred component for low-power and fast-switching applications in the future.

## Data Availability

The data and analysis results generated in this study are available from the corresponding author on reasonable request.
